# Assessment of Anti-Inflammatory and Antioxidant Effects of *Citrus unshiu* Peel (CUP) Flavonoids on LPS-Stimulated RAW 264.7 Cells

**DOI:** 10.3390/plants10102209

**Published:** 2021-10-18

**Authors:** Adhimoolam Karthikeyan, Hun Hwan Kim, Vetrivel Preethi, Mohammad Moniruzzaman, Ki Ho Lee, Senthil Kalaiselvi, Gon Sup Kim, Taesun Min

**Affiliations:** 1 Subtropical Horticulture Research Institute, Jeju National University, Jeju City 63243, Korea; karthik2373@gmail.com; 2 Research Institute of Life Science and College of Veterinary Medicine, Gyeongsang National University, Gazwa, Jinju 52828, Korea; shark159753@naver.com (H.H.K.); preethivetrivel05@gmail.com (V.P.); 3 Department of Animal Biotechnology, Jeju International Animal Research Center (JIA) and Sustainable Agriculture Research Institute (SARI), Jeju National University, Jeju City 63243, Korea; monir1983@jejunu.ac.kr; 4 Department of Biochemistry and Molecular Biology, College of Medicine, Eulji University, Daejeon 34824, Korea; kiholee@eulji.ac.kr; 5 Department of Biochemistry, Biotechnology and Bioinformatics, Avinashilingam Institute for Home Science and Higher Education for Women, Coimbatore 641028, Tamil Nadu, India; kalaiselvi_bc@avinuty.ac.in

**Keywords:** *Citrus unshiu*, flavonoids, inflammation, lipopolysaccharide, oxidative stress

## Abstract

*Citrus unshiu* is a popular medicinal herb in several Asian countries, in particular South Korea. *C. unshiu* peel (CUP) has several biologically active compounds, including flavonoids. Hence, this research aimed to label the flavonoids from CUP by HPLC-MS/MS analysis and examine their anti-inflammatory and antioxidant potential on LPS-stimulated RAW 264.7 macrophages. A total of four flavonoids (Rutin, naringin, hesperidin, and poncirin) were characterized, and their contents were quantified from CUP. It showed that the naringin is rich in CUP. Further, treatment with the flavonoids at concentrations of 2.5 and 5 μg/mL had no effect on the cell viability of RAW 264.7 macrophages. On the other hand, it decreased the production and expression of inflammatory mediators and pro-inflammatory cytokines such as NO, PGE_2_, TNF-α, IL-1β, iNOS, and COX2 in the LPS-stimulated RAW 264.7 macrophages. In addition, flavonoids treatment inhibited the NF-κB activation by downregulating the p-p65 and p-IκBα proteins expression. Furthermore, reactive oxygen species (ROS) production considerably decreased at the same concentrations while antioxidant enzyme activity increased in the LPS-stimulated RAW 264.7 macrophages. Collectively, our results show that CUP flavonoids have the potential to decrease inflammation and oxidative damage.

## 1. Introduction

Globally, an important health source for people is traditional medicine. It is a major resource of phytochemical compounds with considerable therapeutic activities. Thus, it is essential to evaluate and confirm the therapeutic potential of plants to convince people that use them. Among the different *Citrus* spp. grown in the world, satsuma mandarin (*Citrus unshiu* Marc.) is one of the most popular for characteristics such as juicy and sweet, seedless, easy to peel, and early ripening [1]. Besides, *C. unshiu* is also popular as a medicinal herb by an ancient native community of East Asia and is widely grown in Argentina, Australia, China, Japan, Peru, Russia, Spain, South Korea, South Africa, and Uruguay in semi-warm and warm climates [1,2]. Citrus peels account for nearly half of the fruit body in citrus fruits and are the main waste part used to produce a vast amount of by-products [3,4]. Noticeably, *C. unshiu* peel (CUP) is characterized by the presence of biologically active compounds, including flavonoids, phenolic acids, vitamin C, fiber, and many nutrients compared to its fruits. These compounds are characteristic and prime in *Citrus* spp. that have potent therapeutic effects. Previous studies reported that CUP has the potential to enhance the many digestive dysfunctions (i.e., Tympanites, nausea, vomiting, and dyspepsia), bronchitis, asthma, cardiac and blood circulation-related issues [5,6]. Among the various biological compounds, special attention has been paid to flavonoids, a group of natural polyphenolic compounds, because of their anti-inflammatory and antioxidant activities. Thus, CUP is an attractive and potential raw material used for cosmetics, pharmaceuticals, and functional food.

Recently, researchers have expressed great interest in oxidative stress and the condition of the enormous generation of ROS in the organism. Lipopolysaccharide (LPS) triggers ROS generation by several mechanisms that include activation and induction of NADPH oxidase and downregulation of antioxidant enzymes associated with the clearance of ROS [7]. LPS can provide stimulus oxidative stress mediators and primary to the production of ROS, that prompt cytokine’s production by themselves. Oxidative stress reason for the induction of cytokines by NF-κB [8]. Mounting evidence supports the statement that LPS activation leads to the pro-inflammatory cytokines (TNF-α, IL-1β, and IL-6), inflammatory mediators (NO and PGE_2_) production, and ROS generation that results in the development of many inflammatory diseases [9,10,11]. For the time of infection and the progress of the disease, macrophages have a primary role in the host immune defense system. The uncontrolled penetration of stimulated macrophages in flawed adipose tissue coupled with a tenacious release of ROS, inflammatory mediators, and cytokines contribute to chronic inflammation [12]. Besides, transcription factors (i.e., NF-κB and JNK) also play a major part in this development [13]. It has been found that the NF-κB pathway is linked to the progression of inflammation-associated diseases. Thus, many therapeutic compounds derived from plants aim at the NF-κB signaling pathway that might obstruct inflammation development [14].

CUP and its phytochemical compounds are excellent nutritional dietary supplements or even a promising therapeutic agent. Several studies explored its phytochemical compounds and biological characteristics. However, the therapeutic benefits of *C. unshiu* should be supported with an additional consistent and systematic study. Taking into account the above, the objectives of this research were to (i) characterize the flavonoids from CUP using HPLC-MS/MS, (ii) examine the anti-inflammatory and antioxidant effects of CUP flavonoids on LPS-stimulated RAW 264.7 cells.

## 2. Results

### 2.1. Flavonoids from CUP

**Figure 1:**
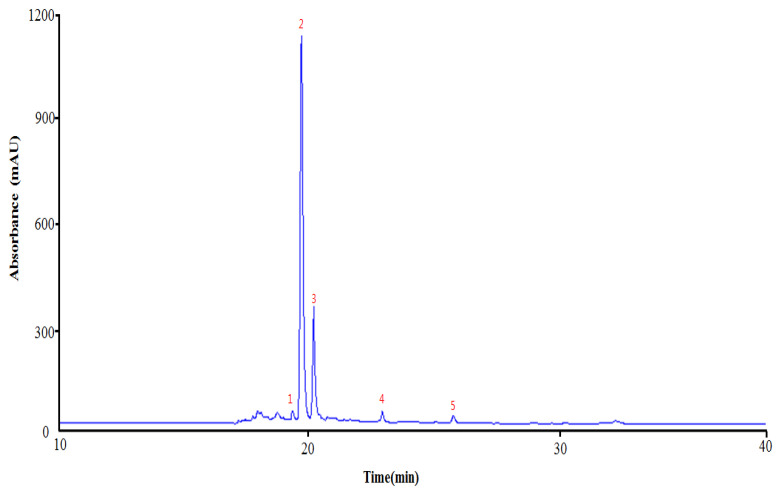
High performance liquid chromatography (HPLC) profile of flavonoids identified from *Citrus unshiu* peel. Note: (1) Rutin, (2) Naringin, (3) Hesperidin, (4) Poncirin, and (5) Unknown.

**Figure 2:**
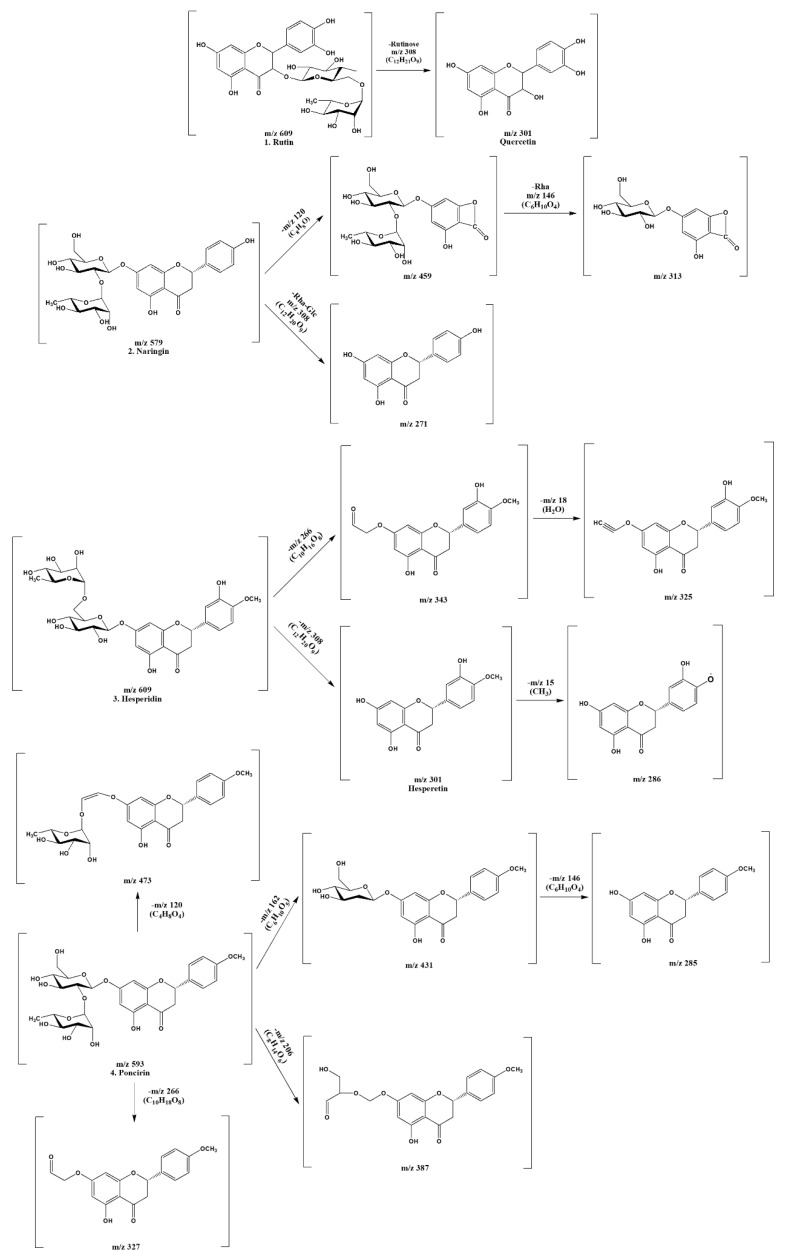
Fragmentation scheme of the flavonoids contained in *Citrus unshiu* peel.

Identification of flavonoid compounds in *C. unshiu* peel was conducted simultaneously using HPLC-MS/MS analysis. The HPLC chromatogram of the *C. unshiu* peel, which was observed at 280 nm, is presented in Figure 1. The structure of the compound identified and the outcome of envisaging the cleavage of the compound based on the LC-MS/MS results are presented in Figure 2 and Table 1. The flavonoid Compound **1** (retention time, t_R_ = 19.25 min) yield [M-H]^−^ at m/z 609, which was fragmented to ions m/z 301 (Quercetin) [M-H-Rutinose (C_12_H_21_O_9_)]^−^ was identified as rutin. Compound **2** (t_R_ = 19.66 min) yield [M-H]^−^ at m/z 579 with additional peaks observed at m/z 459 [M-H-C_8_H_8_O]^−^, m/z 313 (C_13_H_14_O_9_), and m/z 271 [M-H-Rha-Glc] was identified as naringin. Compound **3** (t_R_ = 20.19 min) was identified as hesperidin and showed [M-H]^−^ at m/z 609, which was fragmented to generate an ion at m/z 343 [M-H-C_10_H_16_O_8_]^−^, m/z 325[M-H-C_10_H_18_O_9_]^−^, m/z 301 [M-H-C_12_H_20_O_9_]^−^, and m/z 286 [M-H-C_13_H_23_O_9_]^−^. Compound **4** (t_R_ = 23.09 min) yielded [M-H]^−^ at m/z 593, which was fragmented to ions [M-H-C_4_H_8_O_4_]^−^, [M-H-C_6_H_10_O_5_]^−^, [M-H-C_8_H_14_O_6_]^−^, [M-H-C_10_H_18_O_8_]^−^, and [M-H-C_12_O_20_O_9_]^−^ observed at m/z 473, 431, 387, 327, and 285, respectively. It was identified as poncirin. Further, quantification of the individual flavonoids was performed using calibration curves obtained from structurally related external standards. As presented in Table 2, satisfactory validation data were obtained for the parameters considered. The calibration curve (R_2_) was found to be ≥0.9998. The limits of detection and limits of quantitation were between 0.064–0.927 and 0.192–2.780 mg/L, respectively. The contents of the individual components are listed in Table 2. 

### 2.2. Cell Toxicity of CUP Flavonoids

**Figure 3:**
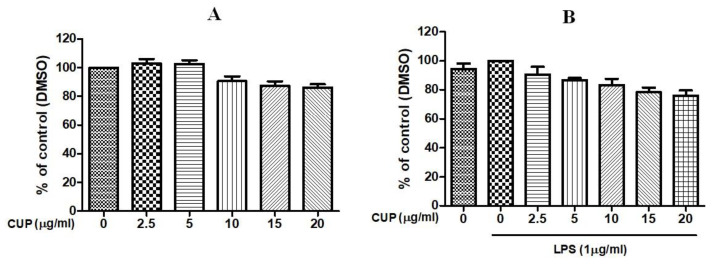
Cytotoxic effect of *Citrus unshiu* peel (CUP) flavonoids on RAW264.7 cells. Different concentrations of CUP (0, 2.5, 5, 10, 15, and 20 μg/mL) were used for cell treatment for 24 h, with or without 1-h pre-incubation of lipopolysaccharide (LPS) (1 µg/mL). Cell viability was measured with the MTT assay. (**A**) Cell viability of non-LPS-stimulated RAW264.7 cells after the treatment with CUP flavonoids at different concentrations. (**B**) Cell viability of LPS-stimulated RAW264.7 cells after the treatment with CUP flavonoids at different concentrations. Note: Data are the mean ± SEM of experiments in triplicate (n = 3).

The cytotoxicity of flavonoids was investigated in RAW 264.7 macrophages to determine the optimal concentration (effective in giving anti-inflammatory and antioxidant effects with minimum toxicity). Figure 3 shows the cell viability at different concentrations (2.5–20 μg/mL) of flavonoids in the presence of LPS (1 μg/mL). The cell viability of RAW 264.7 macrophages was not affected by flavonoids at 2.5 to 10 μg/mL concentrations. Thus, nontoxic flavonoids concentrations 2.5 and 5 µg/mL were selected to determine the anti-inflammatory and antioxidant potential of the flavonoids on LPS-stimulated macrophages.

### 2.3. CUP Flavonoids Inhibit NO Production and iNOS Expression in LPS-Stimulated Macrophages

**Figure 4:**
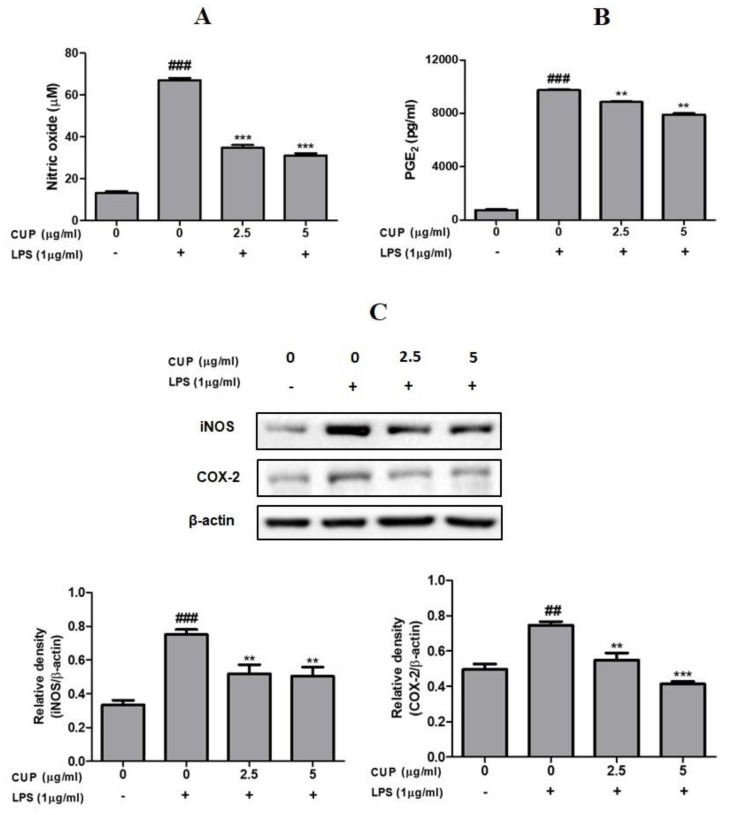
Effect of *Citrus unshiu* peel (CUP) flavonoids on (**A**) nitric oxide (NO) and (**B**) prostaglandin E2 (PGE_2_) production and **(C)** iNOS and COX-2 protein expression levels in LPS-stimulated RAW264.7 cells. NO and PGE_2_ production and iNOS and COX-2 expression levels measured in cells treated at different concentrations of CUP flavonoids (0, 2.5 and 5 μg/mL) for 24 h, with or without 1 h pre-incubation of lipopolysaccharide (LPS) (1 µg/mL). Note: Data are the mean ± SEM of experiments in triplicate (n = 3). ^##^ *p* < 0.01 vs. un treated group; ^###^ *p* < 0.001 vs. untreated group; ** *p* < 0.01 and *** *p* < 0.001 vs. LPS-alone treated group. Relative expression of iNOS and COX-2 was measured and β-actin was used as internal control. Bands expressed in Western blots were measured by densitometry.

NO is produced by iNOS, and it has a main part in the inflammatory response. Thus, to examine the anti-inflammatory effect of flavonoids in LPS-induced macrophages, the NO production was measured by Griess assay (Figure 4). Results showed that following LPS treatment, NO production was considerably increased; but cotreatment with 2.5 or 5 µg/mL of flavonoids decreased the NO production when compared to those who were given LPS only. On the other hand, as shown in Figure 4, LPS-induced RAW 264.7 macrophages increased iNOS expression, whereas cotreatment with 2.5 or 5 µg/mL of flavonoids suppressed iNOS expression. At both concentrations, the iNOS expression level was decreased compared to LPS alone treated cells. Collectively, these results indicate that the flavonoids effectively decrease the NO production by downregulating iNOS expression in the activated macrophages.

### 2.4. CUP Flavonoids Inhibit PGE_2_ Production and COX2 Expression in LPS-Stimulated Macrophages

To further explore the anti-inflammatory effects of flavonoids, PGE_2_ production and COX2 expression were examined in LPS-stimulated macrophages. LPS treatment significantly increased the PGE_2_ production, and it was decreased upon further treatment of flavonoids (2.5 or 5 µg/mL) compared to those treated with LPS alone (Figure 4). Moreover, COX-2 expression at the protein level showed a considerable increase upon treatment with LPS. However, cotreatment with 2.5 or 5 µg/mL of flavonoids inhibited the COX-2 expression and showed a significant decrease compared to those treated with LPS alone (Figure 4). Overall, these findings suggest that the flavonoids efficiently reduce the PGE_2_ production in the activated macrophages by downregulating COX-2 expression. 

### 2.5. Effects of CUP Flavonoids on Pro-Inflammatory Cytokines Production in LPS-Stimulated Macrophages 

**Figure 5:**
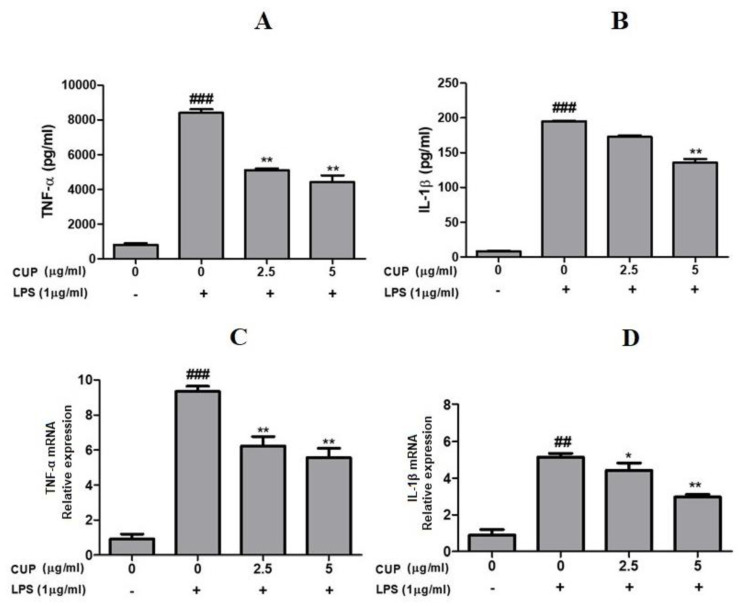
Effect of *Citrus unshiu* peel (CUP) flavonoids on TNF-α and IL-1β levels in LPS-stimulated RAW264.7 cells. TNF-α and IL-1β levels measured in cells treated at different concentrations of CUP flavonoids (0, 2.5, and 5 μg/mL) for 24 h, with or without 1 h pre-incubation of lipopolysaccharide (LPS) (1 µg/mL). (**A**) TNF-α and (**B**) IL-1β levels by ELISA assay. (**C**) TNF-α and (**D**) IL-1β mRNA relative expression by qRT-PCR analysis. Note: Data are the mean ± SEM of experiments in triplicate (n = 3). ^##^ *p* < 0.01 vs. un treated group; ^###^ *p* < 0.001 vs. untreated group; * *p* < 0.05 and ** *p* < 0.01 vs. LPS-alone treated group. Relative expression of TNF-α and IL-1β was measured and β-actin was used as internal control.

To know the effects of flavonoids in LPS-stimulated macrophages, we examined the pro-inflammatory cytokines (TNF-α and IL-1β) by ELISA and qRT-PCR analyses. ELISA analysis showed that following LPS treatment, TNF-α and IL-1β levels were considerably increased; at the same time, cotreatment with 2.5 or 5 µg/mL of flavonoids decreased the TNF-α and IL-1β level compared to those treated with LPS alone (Figure 5). These results were further confirmed by expression analysis. Figure 5 shows that LPS-induced RAW 264.7 macrophages increased TNF-α and IL-1β expression at the mRNA level, whereas cotreatment with 2.5 or 5 µg/mL of flavonoids suppressed TNF-α and IL-1β expression, revealing that flavonoids commendably obstructs the pro-inflammatory cytokines synthesis in activated macrophages.

### 2.6. CUP Flavonoids Disturb the NF-κb Activation and Induce Anti-Inflammatory Effects in LPS-Stimulated Macrophages

**Figure 6:**
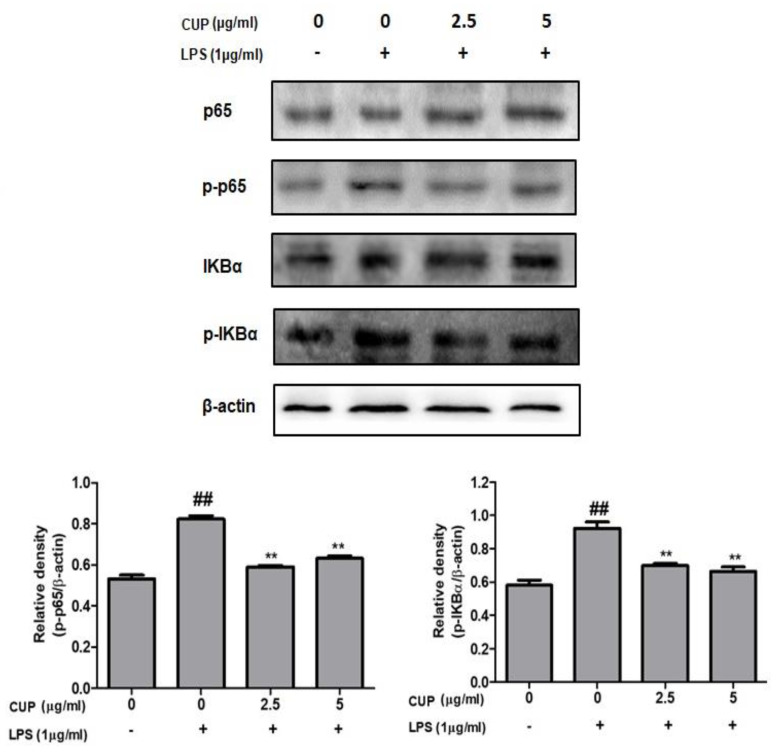
Effect of *Citrus unshiu* peel (CUP) flavonoids on LPS-stimulated p-p65, p65, p-IκBα, and IκBα protein levels in RAW264.7 cells. Cells treated with different concentrations of CUP flavonoids (0, 2.5, and 5 μg/mL) for 24 h, with or without 1 h pre-incubation of lipopolysaccharide (LPS) (1 µg/mL). Relative expression of p-p65/p65, and p-IκBα/IκBα was measured and β-actin was used as internal control. Bands expressed in Western blots were measured by densitometry. Note: Data are the mean ± SEM of experiments in triplicate (n = 3). ^##^ *p* < 0.01 vs. untreated group; ** *p* < 0.01 vs. LPS-alone treated group.

To examine the effects of flavonoids on the NF-κB pathway’s regulation, the expression level of κBα (IκBα) and p65 was analyzed by Western blot analysis. As shown in Figure 6, p-p65 and p-IκBα protein expression were decreased by treatment with the flavonoids, while the p65 and IκBα protein expression continued to be unaffected. The proportion of p-p65/p65 and p-IκBα/IκBα did not increase in the LPS co-treatment with flavonoids. However, it was high in the LPS-alone stimulated macrophages. The findings indicated that flavonoids effectively control the inflammatory response of RAW 264.7 macrophages via inhibiting the NF-κB activation. 

### 2.7. Inhibition of CUP Flavonoids on ROS Production in LPS-Stimulated Macrophages

**Figure 7:**
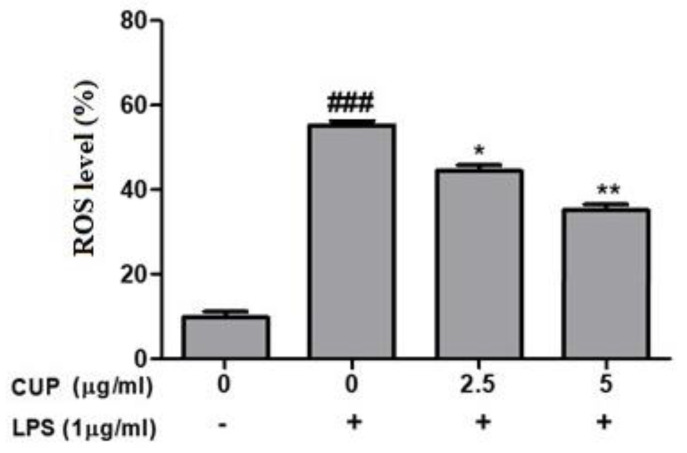
Effect of *Citrus unshiu* peel (CUP) flavonoids on reactive oxygen species (ROS) production in LPS-stimulated RAW264.7 cells. Cells treated with different concentrations of CUP (0, 2.5, and 5 μg/mL) for 24 h, with or without 1 h pre-incubation of lipopolysaccharide (LPS) (1 µg/mL), Note: Data are the mean ± SEM of experiments in triplicate (n = 3). ^###^ *p* < 0.001 vs. untreated group; * *p* < 0.05 and ** *p* < 0.01 vs. LPS-alone treated group.

Further, ROS production was measured to know the flavonoids inhibition on oxidative damage in LPS-stimulated RAW 264.7 macrophages. Results showed that after LPS treatment, ROS production was considerably increased; but cotreatment with 2.5 or 5 µg/mL of flavonoids decreased the ROS production compared to those treated with LPS alone, Figure 7. In addition, antioxidant enzymes (SOD 2 and CAT) proteins expression was considerably increased in LPS-stimulated RAW 264.7 macrophages. It revealed that flavonoids could avert oxidative damage through obstructing ROS production and activating antioxidant enzymes.

## 3. Discussion

In this study, flavonoids in CUP have been characterized and quantified. Further, we have also put the flavonoid’s therapeutical potential to the test by evaluating the anti-inflammatory, and antioxidant effects in LPS-stimulated macrophages. Here, we discuss our major findings with preceding studies.

### 3.1. Flavonoids Identified from CUP

CUP contains a variety of biological compounds, including flavonoids, a group of natural polyphenolic compounds, receiving special emphasis. There is some literature knowledge available for the therapeutic benefits of flavonoids. However, it could be backed up by a more thorough and systematic investigation. This will lead to novel therapeutics with fewer toxic side effects. In this study, four flavonoids were found in the 10–50 min retention time of the chromatograms. Compound **1** (retention time, t_R_ = 19.25 min) yield [M-H]^−^ at m/z 609, that was fragmented to ions m/z 301 (Quercetin) [M-H-Rutinose (C_12_H_21_O_9_)]^−^ was recognized as rutin and correlated with the reports of Charrouf et al. [15]. Compound **2** (t_R_ = 19.66 min) yield [M-H]^−^ at m/z 579 with additional peaks observed at m/z 459 [M-H-C_8_H_8_O]^−^, m/z 313 (C_13_H_14_O_9_), and m/z 271 [M-H-Rha-Glc] was known as naringin [16]. Compound **3** (t_R_ = 20.19 min) was identified as hesperidin and in agreement with the results of Ciric et al. [17] exhibited [M-H]^−^ at m/z 609 that was fragmented to produce an ion at m/z 343 [M-H-C_10_H_16_O_8_]^−^, m/z 325 [M-H-C_10_H_18_O_9_]^−^, m/z 301 [M-H-C_12_H_20_O_9_]^−^, and m/z 286 [M-H-C_13_H_23_O_9_]-. Compound **4** (t_R_ = 23.09 min) yielded [M-H]^−^ at m/z 593, which was fragmented to ions [M-H-C_4_H_8_O_4_]^−^, [M-H-C_6_H_10_O_5_]^−^, [M-H-C_8_H_14_O_6_]^−^, [M-H-C_10_H_18_O_8_]^−^, and [M-H-C_12_O_20_O_9_]^−^ observed at m/z 473, 431, 387, 327, and 285, respectively. It was known as poncirin and coupled with the reports of Shi et al. [18]. Moreover, flavonoids detected in CUP were measured by HPLC-UV chromatograms (284 nm) that matched each reference compound. A calibration curve is generated from a reference compound with structural properties corresponding to those found in our characterization results. The analysis of the individual components showed that, among the flavonoids, naringin was found to be abundant, followed by hesperidin, rutin, and poncirin. Further optimal concentration (in effect in providing anti-inflammatory and antioxidant effects with minimum toxicity) of flavonoids was determined in LPS-stimulated macrophages by MTT assay. It revealed that flavonoids concentrations at 2.5 and 5 μg/mL are nontoxic and could not affect the cell viability. Thus, both 2.5 and 5 μg/mL were chosen and used to study the anti-inflammatory and antioxidant effects of flavonoids on LPS-stimulated macrophages.

### 3.2. Anti-Inflammatory Potential of CUP Flavonoids

Inflammation is known as a protective mechanism of organisms to rid themselves of destructive stimuli. It is normally helpful and classified as either acute or chronic inflammation. Inflammation triggered by harmful microorganisms is mainly mediated by macrophages [19]. LPS stimulated macrophages to cause the release of a number of pro-inflammatory related factors, resulting in damage to tissues and general problems [20]. According to preceding studies, chronic inflammation causes the upsurge of inflammatory mediators (NO, PGE_2_, iNOS, and COX2) and cytokines (TNF-α and IL-1β) [21,22]. iNOS and COX-2 are accountable for alleviating the NO and PGE_2_ production levels, correspondingly. Both play a vital role in the development of inflammatory diseases pathogenesis. In this sense, we have tested the flavonoid’s anti-inflammatory effects in LPS-stimulated RAW 264.7 macrophages. Our results confirmed that flavonoids effectively decreased the NO and PGE_2_ production (Griess and ELISA assays) by downregulating the expression (At protein level) of their synthetic enzymes (iNOS and COX-2). These results are reliable with the reports of Hwang et al. [23] and Han et al. [24], who described that downregulation of iNOS and COX-2 is related to the decrease of NO and PGE_2_ production. Moreover, it suppresses the pro-inflammatory cytokines such as TNF-α and IL-1β levels in LPS-stimulated RAW 264.7 macrophages. It was confirmed by ELISA and expression (At mRNA level) analyses [25,26,27]. Taking together, these findings show that flavonoids effectually recover inflammatory conditions by way of suppressing the excessive production of inflammatory mediators and cytokines in activated macrophages. Many studies have shown that NF-κB is studied as the primary target for regulating or intervening in inflammatory-related genes and enzyme expression [28,29]. It is a key transcription factor associated with inflammatory mechanisms in immune cells. A typical NF-κB activity owing to innumerable causes is known to be involved in the underlying mechanisms of different autoimmune diseases [30]. NF-κB signaling is responsible for producing inflammatory mediators and cytokines by phosphorylating, ubiquitinating, and subsequent proteolytic degradation of NF-κB-bound IκB kinase proteins (IκBα and p65) [31,32]. In this study, the treatment of flavonoids (2.5 or 5 µg/mL) inhibited the IκBα and p65 proteins expression, whereas the whole form of these protein’s expression levels continued unaffected. Further, the proportion of p-p65/p65 and p-IκBα/IκBα was not increased in the co-treatment with flavonoids in LPS-stimulated macrophages. However, it was high in the LPS-alone stimulated macrophages. It is proposed that flavonoids increased the RAW264.7 macrophage’s immune function via keeping p65 and IκBα proteins phosphorylation by regulating the NF-κB pathway.

### 3.3. Antioxidant Potential of CUP Flavonoids

Oxidative stress induced by excessive ROS accumulation causes severe damage to cellular lipids, DNA, and proteins, linked to cancers, inflammatory, cardiovascular, and neurological diseases [33,34,35]. LPS activation of macrophages results in the formation of ROS. In general, a cell’s prime defense response to oxidative stress is related to exogenous and endogenous bases. Of these, endogenous bases included mainly antioxidant enzymes [36]. The reduced ROS production or increased antioxidant enzyme activity are needed to control the oxidative damage. According to a review study by Wang et al. [37] and Jin et al. [38], LPS activation causes oxidative stress via increasing the ROS level and downregulating the expression (At protein level) of the antioxidant enzymes in RAW264.7 macrophages. In this study, flavonoids effectively decreased ROS production via upregulating the antioxidant enzymes (SOD and CAT) expression in LPS-stimulated RAW 264.7 cells proposing that flavonoids have antioxidant effects in the course of macrophage activation. The results were coupled with the earlier findings of Hwang et al. [23] and Lee et al. [39], who reported that reduced ROS production is related to increased antioxidant enzyme activity.

## 4. Materials and Methods

### 4.1. Plant Material, Standards, and Reagents

Matured Satsuma mandarin (*Citrus unshiu)* fruits were collected from the Citrus Genetic Resources Bank, Jeju National University, and Jeju, South Korea. The *Citrus unshiu* fruits were washed with water, and then fruit peel was ground well, lyophilized, and stored at −80 °C for further experiments. The chemicals, standards, and reagents used in this study are provided in supplementary information S1.

### 4.2. HPLC-MS/MS Analysis and Quantification of Flavonoids

The powder of *C. unshiu* peel (100 g) was extracted with 4L of 70% aqueous methanol for 24 hrs (Seven extraction cycles), and then extracts were pooled as well as purified by Buchner funnel. The filtrate was concentrated to 100 mL with the support of a rotary evaporator at 40 °C. The concentrate solution was degraded through hexane and taken out using ethyl acetate. Following three cycles (100 mL × 3), the resulting compound (organic layer) was neutralized and become dry with magnesium sulfate (MgSO4) to yield flavonoid components. HPLC analysis was done to characterize the flavonoids [40]. MS/MS analysis was done in negative mode by LC-MS/MS system (Applied Biosystems, Foster City, CA, USA) with a Turbo V source and a Turbo Ion Spray probe. The mass spectra data were observed in the range m/z 100 to 1000. The amount of flavonoids were determined by LC-UV chromatograms (at 280 nm) using the respective standards viz., rutin, naringin, hesperidin, and poncirin. Five different concentrations (n = 62.5; 125, 250, 500, and 1000 µg/mL) were used to plot the calibration curves, and the contents of the flavonoids were quantified with regard to ratios of peak area by the analyte versus analyte concentrations through a 1/x (x referred as concentration) weighted least squares (n = 5). Flavonoids were quantified using a standard with the same chromophore, and all the samples were repeated three times.

### 4.3. Cell Culture

RAW264.7, mouse macrophage cells (American Type Culture Collection) were used in this study. The cells were cultured in DMEM with 10% heat-inactivated FBS, antibiotics [Penicillin (100 U/mL) and streptomycin (100 μg/mL)], and non-essential amino acid (1%) and maintained in 5% CO_2_ humidified incubator at 37 °C. Cells were allowed to grow and sub-cultured every two days. Further experiments were carried out with cells from passages 5 to 12.

### 4.4. Cell Viability Assay

Cell viability was conducted by a colorimetric assay based on MTT reagent as detailed previously [41]. Briefly, 1 × 10^4^ RAW264.7 cells were seeded onto 96-well plates, and cells were given increasing doses of CUP flavonoids (0, 2.5, 5, 10, 15, and 20 μg/mL) for 24 h at 37 °C, with or without 1-h pre-incubation of 1 µg/mL of LPS. The control and LPS alone groups received the same amount of DMSO. Following incubation, MTT solution (10 µL; 5 mg/mL) was added, and then cells were kept for 4 h at 37 °C in dark condition. Then, the culture medium was removed, each well was added with DMSO, and the absorbance was measured at 590 nm by an HT-microplate spectrophotometer (BioTek Instruments, Inc., Winooski, VT, USA).

### 4.5. Nitric Oxide Determination

The nitrite concentration as an index for NO production was measured in the cell culture based on the Griess reaction following the user guidelines. Raw 264.7 cells (1 × 10^4^ cells/well) were plated in 96-well plates and pre-incubated with or without LPS (1 μg/mL) for 1 h. Further, cells were subjected to treatment with flavonoids (2.5 and 5 µg/mL) for 24 h at 37 °C. After incubation, the spent cell culture (100 μL) was mixed with Griess reagent (100 μL) for 10 min at room temperature with darkness, and then the absorbance was measured at 540 nm. The NO production level was measured by drawing the standard curve of NaNO_2,_ and the fresh culture medium was used as a blank.

### 4.6. Measurement of PGE_2_, TNF-α, and IL-1β Levels

RAW264.7 cells (1 × 10^4^ cells/well) were plated in 96-well plates and treated with or without LPS (1 μg/mL). After one hour, the cells were treated with flavonoids (2.5 and 5 µg/mL) and kept for 24 h at 37 °C. Finally, the spent cell culture was used to analyze the concentration of PGE_2_, TNF-α, and IL-1β by ELISA kits following the user guidelines (Enzo Life Sciences, Farmingdale, NY, USA).

### 4.7. Measurement of Reactive Oxygen Species

RAW264.7 cells (1 × 10^4^ cells/well) were seeded in a 96-well plate, and then the cells were stimulated with or without LPS (1 μg/mL) for 1 h, then treated with flavonoids (2.5 and 5) and kept for 24 h. The treated cells were washed three times with PBS before being incubated in PBS comprising H2DCF-DA (10 µM) at 37 °C for 45 min in dark condition. The fluorescence intensity generated was determined by a fluorescent multiwell plate reader (BioTek Instruments, Inc., Vermont, USA) with an excitation wavelength/emission wavelength range at 485/535 nm and the relative amount of ROS produced was calculated.

### 4.8. RNA Isolation and qRT-qPCR Analysis

RAW 264.7 cells (6 × 10^5^ cells/well) were plated in six-well plates and then stimulated with or without LPS (1 μg/mL) for 1 h. After one hour, the cells were treated with flavonoids (2.5 or 5 µg/mL) and kept for 24 h at 37 °C. Total RNA was extracted by the Trizol reagent (Thermo Fisher Scientific, Inc., Waltham, MA, USA), and the quality of the RNA was confirmed on 1.5% agarose gel by studying the extent to which RNA bands were intact, and the quantification of the RNA was done by the Biospectrometer (Eppendorf, Germany). RNA was converted into cDNA by iScript™ cDNA synthesis kit (Biorad Laboratories, Inc., Hercules, CA, USA) following the user guidelines. Then, cDNA was diluted 10-fold and used as the template for qRT-PCR analysis. Total of 20 μL of PCR mix comprising cDNA (2 μL), primer (1.5 μL), 10 μL of AccuPower^®^ 2X GreenstarTM qPCR Master mix (Bioneer, Daejeon, South Korea), and ddH_2_O (6.5 μL) was used. All reactions were performed in 96-well plates using Bio-Rad CFX 96 (Bio-Rad Laboratories, Inc., Hercules, CA, USA). The primer sequences for TNFα, IL-1β, and β-actin were obtained from Kim et al. [42]. β-actin was used as an internal control to normalize, and transcripts changes were measured using the 2^−ΔΔCT^ method. The experiment was performed in triplicate.

### 4.9. Protein Isolation and Western Blot Analysis

RAW 264.7 cells (6 × 10^5^ cells/well) were plated in six-well plates and then stimulated with or without LPS (1 μg/mL) for 1 h. After one hour, the cells were incubated with 2.5 or 5 µg/mL of flavonoids and kept for 24 h at 37 °C, and then protein was isolated according to the method of Lee et al. [43]. The protein concentration was measured by a protein assay kit (Thermo Fisher Scientific, Inc.), following the user guidelines. Western blot analysis was done following the method described by Saralamma et al. [19]. Briefly, equivalent volumes of protein (~10 µg) were parted using SDS-PAGE (10% gels), and it was transferred into PVDF membranes through TE 77 Semi-Dry Transfer Unit (GE Healthcare Life Sciences). Next, 5% of BSA and 5% of skimmed milk were used to block the blots for 1 h at room temperature. After blocking, membranes were subjected to overnight incubation with primary antibodies of COX-2, iNOS, p65, p-p65, IκBα, p-IκBα, SOD 2 and CAT and β-actin (1:1000 dilutions) at 4 °C. Then, the membranes were washed thrice for 10 min with tris buffered saline with Tween 20 (TBST) and then probed with the suitable antibodies for 3 h at room temperature [44]. Finally, the resulted blots were documented with Clarity™ ECL substrate reagent (Bio-Rad Laboratories, Inc.).

### 4.10. Statistical Analysis

The experimental data shown mean at least three replicates with standard deviation (SD). One-way ANOVA conducted with Duncan’s multiple range tests was used to examine the difference among groups using Minitab software (Minitab., Inc., State College, PA, USA). A *p*-value < 0.05 was considered to show statistical significance.

## 5. Conclusions

CUP flavonoids are promising therapeutic candidates. This study provided additional and systematic evidence for CUP flavonoid’s therapeutic benefits in terms of anti-inflammatory and antioxidant effects. In the future, research on the therapeutical benefits of one or combined with more flavonoids will accelerate the development and utilization of CUP flavonoids. Collectively, the results suggested that CUP flavonoids can treat cancers, inflammatory, cardiovascular, and neurological diseases. This information will further boost the CUP flavonoids in herbal acupuncture medicine.

## Figures and Tables

**Table 1:** HPLC-MS/MS (retention time, m/z fragments) parameters of standards used for identification of flavonoids in *Citrus unshiu* peel (CUP).

Peak. No	Compound	Chemical Formula	Retention Time (Min)	UV Max	Ionization Mode[M-H]^−^	M/Z	MS/MS	Reference
1	Rutin	C_27_H_30_O_16_	19.25	264, 354	[M-H]^−^	609	301	15
2	Naringin	C_27_H_32_O_14_	19.66	282, 336	[M-H]^−^	579	459, 313, 271	16
3	Hesperidin	C_28_H_34_O_15_	20.19	284	[M-H]^−^	609	343, 325, 301, 286	17
4	Poncirin	C_28_H_34_O_14_	23.09	284, 326	[M-H]^−^	593	387, 327, 309, 285	18
5	Un known	-	25.75	345, 268	[M-H]^−^	723	417, 403, 360	-

**Table 2:** Regression data, LODs, LOQs for four external standards and amount of flavonoids from *Citrus unshiu* peel.

S. No	Standards	Calibration Curve	R^2^	LOD (mg/L)	LOQ (mg/L)	Quantity (Mg/Kg)
1	Rutin	y = 11.488x − 90.932	0.9998	0.927	2.780	24.07
2	Naringin	y = 32.386x + 645.38	0.999	0.105	0.315	256.87
3	Hesperidin	y = 32.297x + 166.45	1	0.075	0.225	79.17
4	Poncirin	y = 34.263x + 165.96	1	0.064	0.192	3.94

y, peak area of standard; x, concentration of standard (mg/L); LOD, limit of detection; LOQ, limit of quantitation; RSD, relative standard deviation.

## Data Availability

Not applicable.

## Supplementary Materials

The following are available online at https://www.mdpi.com/article/10.3390/plants10102209/s1, supplementary information S1: Chemicals, standards and reagents.
