# Supplementary material for: Assessment of Anti-Inflammatory and Antioxidant Effects of Citrus unshiu Peel (CUP) Flavonoids on LPS-Stimulated RAW 264.7 Cells
Source: Plants (Basel). 2021 Oct 18;10(10):2209. doi: 10.3390/plants10102209 (PMC8538621; doi:10.3390/plants10102209)
Supplement: Supplementary file 1 [file plants-10-02209-s001.zip › plants-1414386-supplementary.pdf]

Chemicals, Standards and Reagents

Methanol, hexane, rutin, naringin, hesperidin, poncirin, 2,2-diphenyl-1-picrylhydrazyl (DPPH), 2,2′-azino-bis (3-ethylbenzothiazoline-6-sulfonic acid) (ABTS), lipopolysaccharide (LPS), dimethyl sulfoxide (DMSO), 3-(4,5-Dimethyl-2-thiazolyl)-2,5-diphenyl-2H-tetrazolium bromide (Methylthiazolyldiphenyl-tetrazolium bromide, MTT), and ascorbic acid were purchased from Sigma-Aldrich (St. Louis, Missouri, USA). 0.22 μM membrane filter purchased from Merck (Kenilworth, New Jersey, USA). Dulbecco's modified Eagle's medium (DMEM), Fetal bovine serum (FBS), antibiotics (Penicillin/Streptomycin), and non-essential amino acid were purchased from Gibco (Grand Island, New York, USA) and Thermo Fisher Scientific (Waltham, Massachusetts, USA). Griess reagent kit purchased from Promega Corporation (Durham, North Carolina). PGE_2 (Cat. No. ADI-900-001), TNF-α (Cat. No. ADI-900-047), and IL-1β (Cat. No. ADI-900-132A) assay kits were purchased from Enzo Life Sciences (Farmingdale, New York, USA). Pierce™ bicinchoninic acid protein assay kit, skimmed milk, and bovine serum albumin (BSA) were purchased from Thermo Fisher Scientific (Waltham, Massachusetts, USA). The primary antibodies of COX-2 (Cat. No. 12282S; 1:1,000), iNOS (Cat. No. 13120S; 1:1,000), p65 (cat. no. 8242S; 1:1,000), p-p65 (cat. no. Ser536; 3033S; 1:1,000), IκBα (cat. no. 4812S; 1:1,000), p-IκBα (Ser32; cat. no. 2859S; 1:1,000), β-actin (cat. no. 3700S, 1:10,000) were purchased from Cell Signalling Technology (Danvers, Massachusetts, USA). The secondary antibodies (anti-rabbit A120-101P and anti-mouse A90-116P,) were purchased from respectively, Bethyl Laboratory, Montgomery, Texas, USA.
